# Postoperative communicating hydrocephalus following glioblastoma resection: Incidence, timing and risk factors

**DOI:** 10.3389/fonc.2022.953784

**Published:** 2022-09-12

**Authors:** Lisa S. Hönikl, Nicole Lange, Melanie Barz, Chiara Negwer, Bernhard Meyer, Jens Gempt, Hanno S. Meyer

**Affiliations:** Department of Neurosurgery, Klinikum rechts der Isar, School of Medicine, Technical University of Munich, Munich, Germany

**Keywords:** glioblastoma, hydrocephalus, cerebrospinal fluid shunt, risk factors, postoperative

## Abstract

**Introduction:**

Glioblastoma (GBM) is the most common malignant primary brain tumor. Treatment includes maximally safe surgical resection followed by radiation and/or chemotherapy. However, resection can lead to ventricular opening, potentially increasing the risk for development of communicating hydrocephalus (CH). Complications such as rebleeding and infection may also lead to CH and, eventually, the need for cerebrospinal fluid (CSF) diversion surgery. In this study, we evaluated the incidence of different types of hydrocephalus and potential risk factors for the development of CH following glioblastoma resection.

**Methods:**

726 GBM patients who underwent tumor resection at our department between 2006 and 2019 were analyzed retrospectively. Potential risk factors that were determined for each patient were age, sex, tumor location, the number of resection surgeries, ventricular opening during resection, postoperative CSF leak, ventriculitis, and rebleeding. Uni- as well as multivariate analyses were performed to identify associations with CH and independent risk factors.

**Results:**

55 patients (7.6%) needed CSF diversion surgery (implantation of a ventriculoperitoneal or ventriculoatrial shunt) following resection surgery. 47 patients (6.5%) had CH, on median, 24 days after the last resection (interquartile range: 17-52 days). 3 patients had obstructive hydrocephalus (OH) and 5 patients had other CSF circulation disorders. Ventricular opening (odds ratio (OR): 7.9; p=0.000807), ventriculitis (OR 3.3; p=0.000754), and CSF leak (OR 2.3; p=0.028938) were identified as significant independent risk factors for the development of post-resection CH. Having more than one resection surgery was associated with CH as well (OR 2.1; p=0.0128), and frontal tumors were more likely to develop CH (OR 2.4; p=0.00275), while temporal tumors were less likely (OR 0.41; p=0.0158); However, none of those were independent risk factors. Age, sex, or rebleeding were not associated with postoperative CH.

**Conclusion:**

Postoperative CH requiring CSF shunting is not infrequent following GBM resection and is influenced by surgery-related factors. It typically occurs several weeks after resection. If multiple risk factors are present, one should discuss the possibility of postoperative CH with the patient and maybe even consider pre-emptive shunt implantation to avoid interruption of adjuvant tumor therapy. The incidence of CH requiring shunting in GBM patients could rise in the future.

## Introduction

Glioblastoma is the most common malignant primary brain tumor. It represents both the most aggressive and most common type of glioma (1, 2). Standard therapy entails maximal safe tumor resection and adjuvant radiation therapy with concurrent and adjuvant temozolomide (3, 4). Prognosis is poor with a median overall survival (OS) of 15 months, and with one of the lowest survival rates of any neoplasm, it has been regarded as one of the deadliest of diseases (2, 5). However, novel therapeutic regimes, the identification of molecular tumor subtypes, and improved surgical strategies have led to a gradual increase in overall survival (4) in the past decade (6–11). For example, two-year survival rates have increased from ca. 10% (3, 12, 13) to ca. 50 or even 75% in subgroups of GBM patients (10, 11). Consequently, more patients undergo multiple treatment cycles including resection surgeries, possibly leading to an increase in treatment-related complications and/or disease-related long-term complications, such as hydrocephalus.

Hydrocephalus is the abnormal accumulation of cerebrospinal fluid (CSF) within the ventricular system. Communicating hydrocephalus (CH) is thought to be a consequence of decreased absorption or, in rare cases, of overproduction, and obstructive hydrocephalus (OH) is due to physical obstruction (14, 15). In GBM, OH mainly arises from tumor mass effect, e.g., when the foramen Monroi, the third or fourth ventricle or the aquaeductus cerebri are obstructed. While OH can occur at any time during the course of the disease, CH typically develops in patients who already underwent treatment like surgical resection or radiation (16, 17). Hydrocephalus in GBM patients can lead to massive symptoms associated with increased intracranial pressure like nausea, vomiting, headache, cognitive decline, reduced vigilance and, eventually, death. Apart from tumor resection in OH, the management of hydrocephalus in GBM patients includes external ventricular drainage and ventriculoperitoneal (VP) or ventriculoatrial (VA) shunting (18).

In adults, the incidence of CH following GBM resection appears to be lower than 10% (16, 17, 19, 20). Reported risk factors are ventricular opening during tumor resection (16), leptomeningeal tumor spread (17), and the number of pre-shunt craniotomies (21).

The aim of this study was to investigate the incidence and timing of occurrence of hydrocephalus and to identify risk factors for the development of CH requiring permanent CSF diversion following GBM resection based on a retrospective analysis of all GBM resections at our institution between 2006 and 2019.

## Materials and methods

### Study design, patient selection

We retrospectively analysed a series of 726 patients who underwent glioblastoma resection surgery at the Department of Neurosurgery at the Klinikum rechts der Isar of the Technical University of Munich between 2006 and 2019. Histologically, there were 710 glioblastomas and 16 gliosarcomas; all were WHO grade IV (2).

### Treatment

All patients underwent microsurgical resection aiming at maximal safe tumor removal based on the recommendation by an interdisciplinary tumor board. Standard procedure at our institution includes transcranial magnetic stimulation – based preoperative functional language and motor mapping, intraoperative neuromonitoring, and 5-ALA-guided resection. The placement of an external ventricular drain following resection surgery was not standard procedure, including cases with ventricular opening. Post-resection adjuvant treatment was based on tumor board recommendations and included radiation and/or chemotherapy and/or tumor-treating fields if indicated according to current treatment standards (3, 10). Reresection was defined as resection of recurrent glioblastoma. Additional resection following intraoperative or early (i.e., within 48 hours) postoperative MRI was not defined as reresection. The indication for reresection was based on imaging (MRI and in select cases additional PET indicating locally recurrent vital tumor) and the clinical condition of the patient. In select cases, non-locally recurring tumor was also operated on based on individual circumstances (e.g., symptomatic multi-focal recurrence or circumscribed distal recurrence).

### Hydrocephalus

The diagnosis of hydrocephalus was based on radiographic assessment (CT and/or MRI showing an increase in ventricle size over the course of treatment that was not associated with atrophy and/or signs of CSF diapedesis) and clinical symptoms like headache, cognitive impairment, urinary incontinence, and/or papilledema. Hydrocephalus types were defined as communicating (i.e., CSF circulates freely within and can exit the ventricles) or non-communicating/obstructive (i.e., CSF is prevented from exiting the ventricles based on a mechanical obstruction of CSF flow, e.g., at the foramen Monroi, the aquaeductus cerebri and/or the fourth ventricle due to the mass effect of a tumor and/or its edema or to a blood clot). We also identified cases with other types of CSF circulation disorder (CSF entrapment within the resection cavity, subdural hygromas).

In some cases, hydrocephalus was transient (e.g., obstructive hydrocephalus due to temporary resection-related edema). Acute hydrocephalus was typically treated with temporary placement of an external ventricular drain. Permanent hydrocephalus (i.e., CH, or OH with permanent obstruction) was treated with permanent CSF diversion surgery (ventriculoperitoneal or ventriculoatrial shunting). The indication for permanent CSF diversion surgery represents the event that determined whether a patient was regarded to have developed a type of hydrocephalus for the purposes of this study.

### Patient characteristics

Our database comprises demographic and clinical parameters including sex, age, number of tumor resections, adjuvant treatment modalities, tumor histology, tumor location, ventricular opening during resection, and postoperative complications like ventriculitis, CSF leak, and rebleeding. Ventriculitis was diagnosed based on laboratory analyses of CSF (elevated cell count, elevated lactate, low glucose) and/or positive CSF culture. Rebleeding was defined as a postoperative bleeding seen on CT and/or MRI that required operative intervention. Any symptomatic rebleeding as well as rebleeding with a mass effect led to the indication for revision.

### Statistical analysis

Statistical analyses were performed using R 3.2 (R Core Team, www.r-project.org). Associations between variables were analysed using Chi-squared tests. To identify independent risk factors for hydrocephalus, logistic regression analysis was done. For all analyses, a difference with an error probability of less than 0.05 was considered statistically significant. Descriptive statistics for demographic variables were generated with means and standard deviations (SDs) or medians with interquartile ranges (IQRs) as appropriate.

## Results

### Patient characteristics

**Figure 1:**
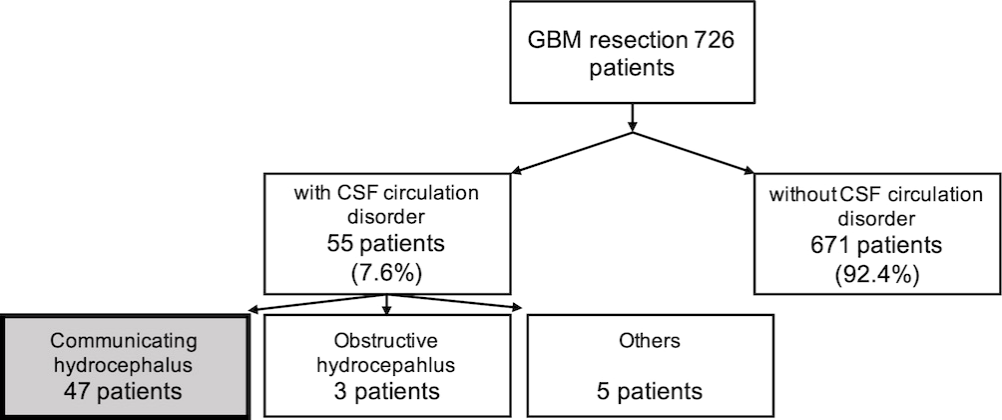
Incidence of different types of CSF circulation disorders in 726 GBM patients who underwent tumor resection between 2006 and 2019. 55 patients developed postoperative CSF circulation disorders, corresponding to an incidene of 7.6%. Of these, 47 had communicating hydrocephalus (CH; 6.5%), 3 had obstructive hydrocephalus (OH; 0.4%), and 5 had other types of CSF circulation disorders (such as hygroma or ventricular entrapment; 0.7%).

We included all 726 patients who underwent resection of a histologically identified GBM at our institution between 2006 and 2019 (**Figure 1**). On average, patients were 64 years old (**Table 1**). 62% were male. The vast majority of tumors was supratentorial (31% frontal or mostly frontal, 26% temporal or mostly temporal, 13% parietal or mostly parietal, 2% occipital or mostly occipital, and 27% affected more than one lobe).

**Table:** Table 1 Characteristics of GBM patients with and without CSF circulation disorders.

	All	No CSF circulation disorder	CH	Other
**N of patients**	726	671	47	8
**Median Age [years (min-max)]**	64 (10-90)	63 (10-88)	60 (20-90)	73 (38-78)
**Male sex [n (%)]**	499 (69%)	415 (62%)	30 (64%)	4 (50%)
**Tumor Localization [n(%)]**				
frontal	224 (31%)	202 (30%)	21 (45%)	1 (13%)
temporal	189 (26%)	182 (27%)	4 (9%)	3 (38%)
parietal	94 (13%)	86 (13%)	8 (17%)	0 (0%)
occipital	18 (2%)	18 (3%)	0 (0%)	0 (0%)
more than one lobe	197 (27%)	180 (25%)	14 (30%)	3 (38%)
infratentorial	4 (1%)	3 (0%)	0 (0%)	1 (13%)

CH patients did not differ from those without hydrocephalus with regards to age or sex, but tumor locations differed significantly (one way ANOVA, p = 0.0485): there were more frontal and less temporal tumors in patients with CH.

Communicative hydrocephalus (CH) was by far the most frequent type; Other types were rare (obstructive hydrocephalus, n = 3; hygroma, n = 2; resection cavity entrapment, n = 3).

### The incidence and temporal development of hydrocephalus after glioblastoma resection

**Figure 2:**
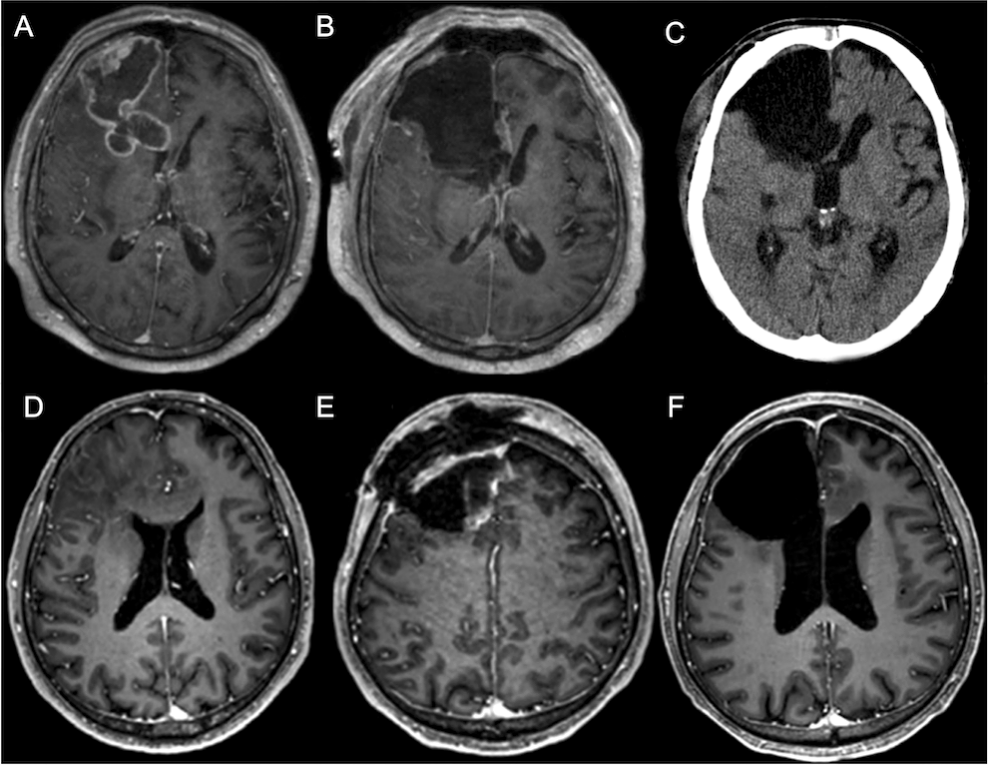
Case illustrations of two GBM patients who developed CH. Patient 1 **(A–C)** was a 60-year-old male with a right frontal GBM (**A**, preoperative MRI/post-gadolinium t1). The right lateral ventricle was opened during resection (**B**, postoperative MRI). One month after surgery, the patient presented with headache, nausea and vomiting. A CT scan showed a progressive ventriculomegaly, consistent with CH **(C)**. Patient 2 **(D–F)** was a 58-year-old male with a right frontal GBM (**D**, MRI/post-gadolinium t1). Some days after tumor resection including ventricular opening, a subcutaneous fluid collection occurred, consistent with a CSF fistula (**E**, MRI/post-gadolinium t1). Some weeks after revision surgery, the patient complained of headache and vomiting, and CH was diagnosed (**F**, MRI/post-gadolinium t1). Both patients received a VP shunt.

55 patients (7.6%) developed CSF circulation disorders requiring CSF diversion after tumor resection (**Figure 2**), most of which had CH (47 patients, 6.5%; for a comparison with patients who did not develop hydrocephalus, see **Table 1**). Eight patients had other CSF circulation disorders (three patients had OH and five patients had resection cavity entrapments or subdural hygromas).

CSF shunting for CH was, on average, performed 24 days (median) after the last resection surgery (OH: 17 days; entrapments/hygromas: 46 days; all types of hydrocephalus: 24 days). The time from resection to shunting for CH was variable (IQR: 17-52 days). It did not depend on the number of resection surgeries (data not shown).

### Risk factors for the development of CH after glioblastoma resection

We investigated potential risk factors for the development of CH following GBM resection. Age and sex were not associated with CH. The distribution of tumor locations differed significantly between patients with CH and those without hydrocephalus (p=0.0485, one way ANOVA): there were more frontal and less temporal tumors in patients with CH (cf. **Table 1**). Both locations were significantly associated with CH (univariate analysis; frontal: OR 2.4 [95% CI 1.4-4.3], p=0.00275); temporal: OR 0.4 [0.2-0.8], p=0.0158), but did not turn out to be independent risk/protecting factors in multivariate analysis (**Table 2**).

**Table 2:** Potential risk factors for the entire cohort and patients with different types of postoperative CSF circulation disorders (CH: communicative hydrocephalus; Other: obstructive hydrocephalus, hygroma, resection cavity entrapment).

	Number of Patients				Statistical Analysis			
	All	CSF Circulation Disorder			Univariate		Multivariate	
		None	CH	Other	OR (CI)	p	OR (CI)	p
Ventriculitis	80 (11.0%)	60 (8.9%)	17 (36.2%)	3 (37.5%)	6.0 (3.3-11.1)	0.00010	3.3 (1.6-6.6)	0.000754
Ventricular Opening	525 (72.3%)	473 (70.5%)	45 (95.7.%)	7 (87.5%)	7.1 (2.2-23.0)	0.00110	7.9 (2.7-33.4)	0.000807
CSF Leak	72 (9.9%)	55 (8.2%)	15 (31.9%)	2 (25.0%)	4.6 (2.4-8.8)	0.00010	2.3 (1.1-4.8)	0.028938
Rebleeding	57 (7.9%)	49 (7.3%)	8 (17.0%)	0 (0.0%)	2.2 (0.9-5.0)	0.05300	2.2 (0.8-5.1)	0.087490
>1 Tumor resection	322 (44.4%)	290 (43.2%)	28 (59.6%)	4 (50.0%)	2.1 (1.1-3.7)	0.01280	1.6 (0.8-2.9)	0.164410
Frontal Tumor	310 (42.7%)	276 (41.1%)	32 (68.1%)	2 (25.0%)	2.4 (1.4-4.3)	0.00275	2.0 (1.0-4.4)	0.056452
Temporal Tumor	231 (31.8%)	222 (33.1%)	5 (10.6%)	4 (50.0%)	0.4 (0.2-0.8)	0.01580	0.5 (0.2-1.4)	0.188536

Patients who developed CH more often had ventricular opening, ventriculitis, CSF leak, more than one tumor resection and rebleeding than those without. They were more likely to have a frontal tumor and less likely to have a temporal tumor. All these factors except for rebleeding were significantly associated with CH (univariate analysis). Ventricular opening, ventriculitis, and CSF leak were identified as independent risk factors (multivariate analysis). Frontal tumors also include those that affected more than one lobe but not the temporal lobe, and temporal tumors also include those that affected more than one lobe but not the frontal lobe.

We also investigated potential surgery-related risk factors (**Table 2**). Ventricular opening, postoperative ventriculitis, CSF leak, more than one tumor resection and rebleeding were more frequent in patients with CH than in those without hydrocephalus, and except for rebleeding, all were significantly associated with CH.

Ventricular opening had occurred in 70.5% of patients without hydrocephalus and in 95.7% of those who developed CH. It was identified as an independent risk factor for the development of post-resection CH (multivariate analysis; OR 7.9 [2.7-33.4], p=0.000807). On average, CSF shunting was performed 43 days (median; IQR: 18-121 days) after the last resection in these patients.

Postoperative ventriculitis also turned out to be an independent risk factor (OR 3.3 [1.6-6.6], p=0.000754). It was found in 8.9% of patients without hydrocephalus and in 36.2% of patients with CH. In this group, CSF shunting was performed 28 (14-101) days after the onset of ventriculitis and 59 (27-117) days after the last resection.

Postoperative CSF leak was identified as another independent risk factor (OR 2.3 [1.1-4.8], p=0.028938). It had occurred in 8.2% of patients without hydrocephalus and 31.9% of those who developed CH. These patients had CSF shunting surgery 11 (6-50) days after resection surgery.

Rebleeding had occurred in 7.3% of patients without hydrocephalus and in 17.0% of those who developed CH. Shunting was performed 12 (8-23 days) after the last tumor resection in these patients. However, rebleeding was neither significantly associated with CH (univariate analysis; OR 2.2 [1.0-5.0], p=0.053) nor found to be an independent risk factor (multivariate analysis; OR 2.2 [CI 0.8-5.1], p=0.087490).

### Multiple resections

On average, GBM patients without hydrocephalus had 1.6 tumor resections (range 1-6), and those who developed CH had 1.9 (1-5) resections. Tumor re-resections were performed in 43.2% (290/671) of patients without postoperative hydrocephalus and in 59.6% (28/47) of patients who developed CH. Having more than one resection was significantly associated with CH (p=0.0128, OR 2.1, CI 1.2-3.7), but it was not found to be an independent risk factor (OR 1.6 [0.8-2.9], p=0.164410; **Table 2**).

The majority of patients with re-resections had only one re-resection, but there were patients with multiple resections in patients without hydrocephalus and in patients with CH (one re-resection: 32.2% of all patients without hydrocephalus vs. 48.9% of all patients with CH; two re-resections: 8.8 vs. 2.1%; three re-resections: 1.8 vs. 2.1%; four re-resections: 0.3 vs 6.4%; five re-resections: 0.3% vs none).

**Figure 3:**
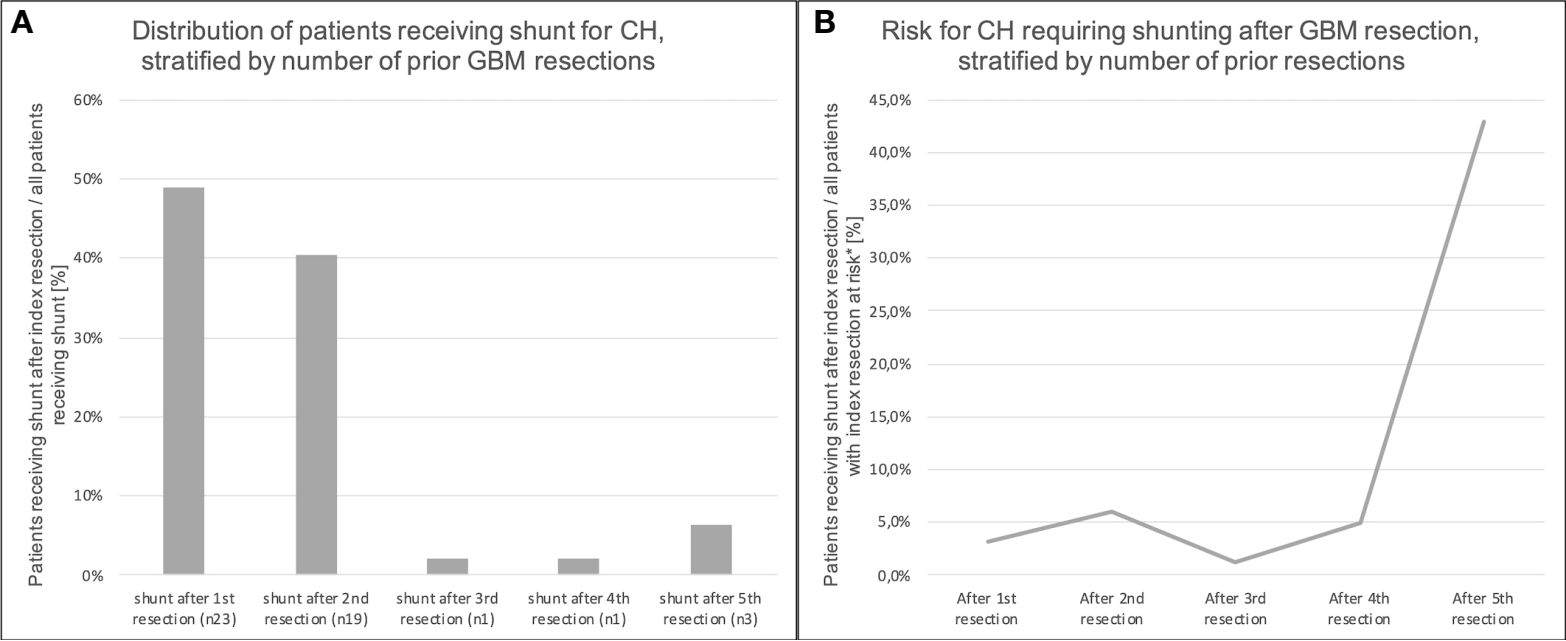
Relation of the number of GBM resections and shunting for CH. **(A)** illustrates the relative frequencies of patients receiving a shunt for CH, stratified by the number of resection surgeries prior to shunting. Half of the patients received their shunt after the first resection. The absolute risk for the development of CH after GBM resection increased with the second resection, but decreased with the third. After the fourth and fifth resection, it increased again **(B)**. * “at risk” = patients who do not have a shunt already.

Half of all patients with postoperative CH were diagnosed after the first resection surgery, the other half after re-resections (second to fifth resection, **Figure 3A**). The risk for the development of CH after the first four tumor resection surgeries varied between 1.5 and 6% and increased to 43% after the fifth tumor resection (**Figure 3B**).

Each of the independent risk factors ventricular opening, CSF leak and ventriculitis, if present in patients with CH and multiple resections, occurred only once in the course of disease, and mostly occurred during the last pre-shunting resection: 27 of 45 patients with CH and ventricular opening had multiple resections. In this group, ventricular opening had occurred during the last pre-shunting resection surgery in 80%. 11 of 15 patients with CH and postoperative CSF leak had multiple resections. CSF leak had occurred after the last pre-shunting resection surgery in 73.3% of this group. 12 of 17 patients with CH and postoperative ventriculitis had multiple resections. In this group, ventriculitis had occurred after the last pre-shunting resection surgery in 82.4%.

### Risk for shunting over time

**Figure 4:**
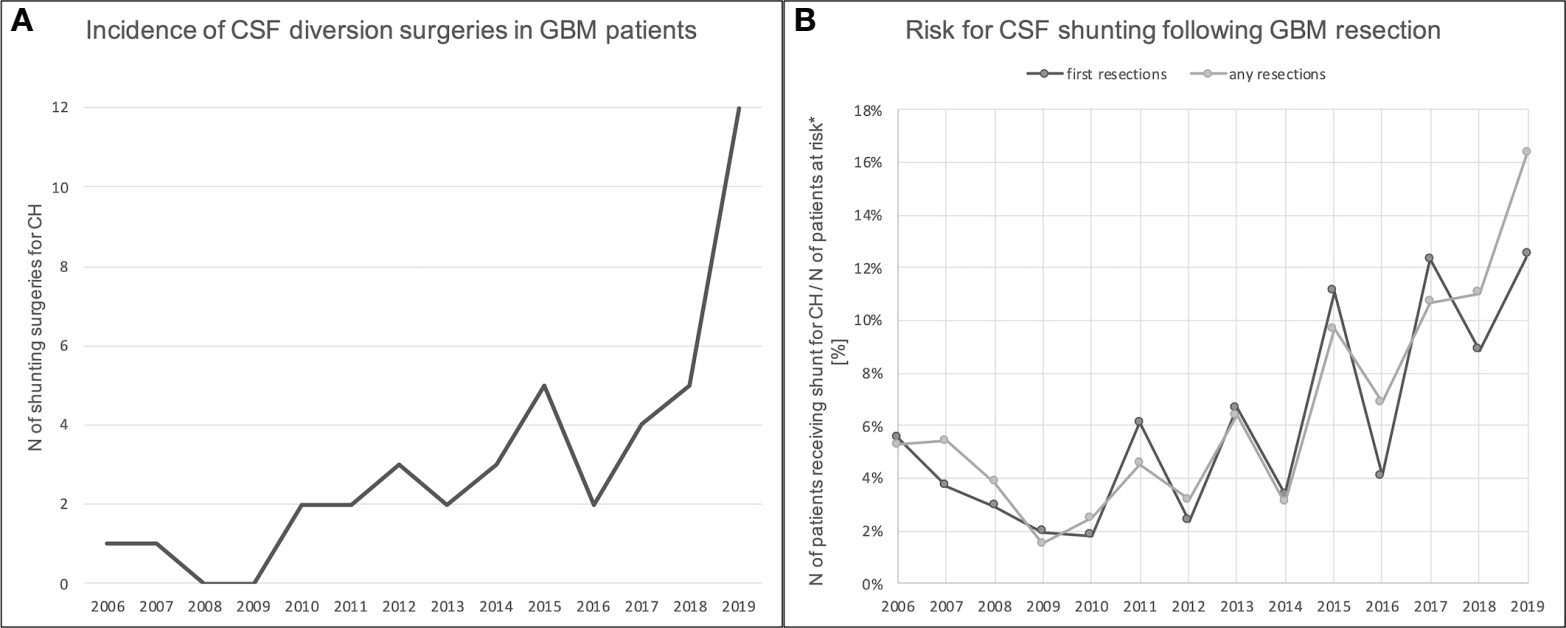
Incidence of and risk for CSF shunting for CH following tumor resection in GBM patients between 2006-2019. **(A)** shows the total number of shunting surgeries for CH in GBM patients per year. There was an increasing trend over the years. **(B)** At the same time, there was an increasing trend in the risk for the development of CH following GBM resection (both first resections and re-resections). *at risk = patients who do not have a shunt already.

There were increasing trends in both the absolute number of CSF shunting surgeries for CH as well as in the risk for the development of CH following GBM resection (**Figure 4**). The latter was true for patients undergoing first resections as well as for those with any instance of resection. Of note, the risk for shunting due to CH may be underestimated for patients resected in recent years given the shorter follow up.

## Discussion

### The incidence of communicating hydrocephalus after glioblastoma resection

We present one of the largest series of glioblastoma patients investigating the incidence and temporal development of post-resection hydrocephalus and risk factors for the development of CH to date.

We found that 7.5% of GBM patients developed CSF circulation disorders requiring CSF diversion surgery in the course of their disease following resection; 6.5% developed CH. This is substantially higher than the 2.1% found in another large recent series (22), but well within the range of several previous smaller series reporting the incidence of hydrocephalus in GBM. In 2003, a study identified 5 of 50 patients (10%) with supratentorial glioblastoma who developed hydrocephalus in the course of disease (19). Montano et al. (16) evaluated GBM patients from 2005 to 2009 and reported that 5.6% (7 of 124) developed postoperative hydrocephalus. Another study with 151 GBM patients from 2007 to 2011 reported 11 patients (7.3%) with postoperative hydrocephalus (17). Castro et al. (21) reported 64 out of 841 GBM patients (8%) who underwent shunting between 2004 and 2014 at their department.

### The temporal development of hydrocephalus after glioblastoma resection

We found that hydrocephalus typically developed within several weeks after tumor resection. This was true for first resections and re-resections. CSF shunting for CH was performed, on average, three weeks after the last resection surgery; the majority had shunting between two and seven weeks. This means that these patients had usually been discharged from neurosurgical inpatient treatment already and had to be readmitted for another surgery, possibly interrupting or delaying adjuvant treatment.

### Risk factors for the development of communicating hydrocephalus after glioblastoma resection

The size of our series enabled us to identify several independent risk factors for the development of post-resection CH, some of which were not investigated or reported in previous studies (22). All of them were surgery-related; basic patient factors, such as gender or age, did not appear to be relevant, which is in accordance with the earlier reports mentioned above.

We found that postoperative ventriculitis is an independent risk factor for post-resection CH in GBM patients (OR 3.3). In our cohort, 11.0% (80 out of 726) of patients had postoperative ventriculitis, 36.2% of which developed CH. The risk for post-operative ventriculitis/meningitis after craniotomy in general has been reported as 0.3%–8.6% in previous studies, and it is well known that ventriculitis/meningitis can lead to CH (23–26). However, to our knowledge, our study is the first to show that postoperative ventriculitis is an independent risk factor for the development of CH following GBM resection. The relatively high ventriculitis rate in our series could be explained by the high proportion of patients with multiple resections, chemotherapies and radiation, which have been shown to increase the risk for infection (27–29).

Postoperative CSF leak is a well-known and frequent complication after cranial surgery with a prevalence of 3-11% (30, 31). Inadequate dura closure as well as increased CSF pressure can contribute to CSF leaks, and postoperative radiation and chemotherapy may also play a role by preventing adequate dural regrowth (32, 33). In this study, 9.9% (72 out of 726) of patients had a CSF leak, consistent with previous reports (30, 31, 34). 31.9% of these patients developed CH, and CSF leak was identified as another independent risk factor for CH with an OR of 2.3. To our knowledge, this is the first study showing this relationship. Since it was found to be an independent risk factor, this cannot be explained by an increased risk for ventriculitis in patients with CSF leak. It is more likely that CSF leak can be an early sign of CH indicating that CH is already developing in these patients.

While a recent study of 200 GBM patients reported no effect of ventricular opening on the incidence of postoperative hydrocephalus (35), several others found that it contributes to the development of CH, possibly due to increased CSF protein levels or CSF dissemination of other toxic materials (16, 17, 22). This was confirmed by our data: 70.5% of the entire cohort vs. 95.7% of those with CH had ventricular opening, and ventricular opening was an important independent risk factor for CH with an OR of 7.9. It can be assumed that ventricular spread of tumor debris, parenchyma, and blood, as well as their degradation products, mediate CH in these patients. Consequently, even though the main goal in GBM resection surgery is cytoreduction, ventricular opening should be avoided, if possible, to lower the risk for post-resection CH. If unavoidable, one should attempt to cover the ventricular opening during resection with neurosurgical patties, and in some cases, it may even be possible to permanently re-close opened ventricles at the end of resection, e.g., by using sponge sealant patches. The perioperative insertion of intraventricular drains might also reduce the risk for post-resection CH, e.g., by diverting toxic materials during the initial postoperative phase. The effectiveness of such measures should be evaluated prospectively in future studies.

Given the substantial effect of ventricular opening on the risk for the development of CH, it is not surprising that frontal tumor location is significantly associated with CH as well. Ventricular opening occurs frequently in those tumors, and entry into the frontal horn of a patient in supine position may cause massive dissemination of toxic material into the ventricular system. This is different from temporal tumors that were significantly less likely found in patients with CH: even though the temporal horn may be opened, it often collapses during resection and/or the head is rotaded laterally, so that toxic influx into the ventricular system may be limited in these cases.

It has been shown that the number of craniotomies plays a significant role in the development of post-resection CH (16). In this study, we found that the number of tumor resection surgeries is significantly associated with the risk for the development of CH. However, it was not an independent risk factor, possibly because it just reflects the accumulation of actually independent risk factors, such as ventricular opening or ventriculitis.

Even though rebleeding was more frequent in patients with CH, there was no significant association, and rebleeding was not an independent risk factor.

Our study was focused on surgery-related risk factors. However, there might be more relevant events that we did not investigate, such as revision surgery for reasons other than rebleeding or CSF leak, e.g., for brain abscess. In fact, we found that “having had any revision surgery” is a significant risk factor in univariate analysis (p<0.0001, OR 4.98). However, we decided not to include this variable in our multivariate analysis in order to be able to analyse more specific events, such as rebleeding or CSF leak, that would otherwise overlap with “any revision surgery”. Ideally, one should investigate all specific types of revision surgery individually, but that would require a much larger sample size than ours. There are further possible risk factors that have been investigated by others. E.g., it has been hypothesized that leptomeningeal tumor spread or intraventricular dissemination can lead to CSF circulation disorders (19, 36), possibly associated with the increased risk following ventricular opening we found; other studies, however, found no such evidence (16, 17, 21). Radiation has also been suggested as a risk factor for the development of hydrocephalus (37, 38). Finally, CH may also be a late consequence of the tumor itself that could occur more frequently due to longer patient survival, maybe especially in patients with subtotal tumor resection; in our study, while we did not analyse the correlation with extent of tumor resection, we found that the risk for CH tended to increase within the last ten years. An overview of studies reporting the incidence and/or risk factors for hydrocephalus following GBM resection is provided by **Table 3**.

**Table 3:** Overview of studies reporting the incidence and/or risk factors for the development of hydrocephalus following GBM resection.

Study	Incidence of Hydrocephalus	Risk factors identified
Montano et al. (16)	5.6%	Ventricular Opening, Number of Craniotomies
Fischer et al. (17)	7.3%	Ventricular Opening
Inamasu et al. (19)	10%	Leptomeningeal/Intraventricular Dissemination
Castro et al. (21)	8%	
El Rahal et al. (22)	2.1%	Ventricular Opening
Hussein et al. (23) Chen et al. (24) De Bonis et al. (25) Oberbauer et al. (26)	–	Ventriculitis
Onda et al. (36)	–	Leptomeningeal/Intraventricular Dissemination

### Changes over time

In our institution, the absolute number of shunt surgeries for CH following glioma resection increased over the years; a clear increase in the risk for the development for CH was not found, but there was an increasing trend in recent years (**Figure 4**). Notably, we might have underestimated the risk in recent years due to limited follow up time. Given our findings, one can expect it to increase in the future: Patients are treated more aggressively, survive longer and accumulate more surgery-related risk factors than in in the past. More lines of adjuvant chemotherapeutic and/or radiation treatment might also add to the risk for the development of CH, and it should not come as a surprise if the number of glioma patients requiring CSF diversion surgery increases in the future.

### Study limitations

The main limitation of our study is that it is a retrospective analysis, potentially leading to, e.g., a loss of patients undergoing CSF diversion surgery elsewhere. The incidence of shunting for CH in our cohort should thus be regarded as a lower-bound estimate. Moreover, while the monocentric nature of our study increases homogeneity, it also potentially introduces bias, such as the relatively high rate of re-resected patients in our cohort. Finally, there are patient-related and surgery-related potential risk factors that we did not analyze, such as tumor burden, preoperative comorbidity burden, molecular tumor properties, or revision surgery for reasons other than rebleeding or CSF leak.

### Conclusion

Postoperative CH requiring shunting affects a relevant proportion of patients following GBM resection. CH typically manifests a few weeks after resection and requires additional neurosurgical treatment for shunt implantation, potentially delaying adjuvant radiation and/or chemotherapy treatment. Several surgery-related events increase the risk for the development of CH. This should be discussed with the patient ahead of surgery. If multiple risk factors are present early after resection (i.e., ventricular opening, ventriculitis, CSF leak), one should discuss the possibility of postoperative CH with the patient in detail and maybe even consider pre-emptive shunt implantation given the established clinical benefit of shunting and in order to avoid early neurosurgical readmission. The number of GBM patients requiring shunting for CH might increase in the future.

## Data availability statement

The raw data supporting the conclusions of this article will be made available by the authors, without undue reservation.

## Ethics statement

The studies involving human participants were reviewed and approved by Ethics committee of the Klinikum rechts der Isar (Ref. 5625/12). Written informed consent from the participants’ legal guardian/next of kin was not required to participate in this study in accordance with the national legislation and the institutional requirements.

## Author contributions

Conceived the study: HM. Curated data: LH and MB. Analyzed data: NL, LH and HM. Wrote the manuscript: HM and LH with input from all authors. Supervised the study: HM. All authors contributed to the article and approved the submitted version.

## Conflict of interest

The authors declare that the research was conducted in the absence of any commercial or financial relationships that could be construed as a potential conflict of interest.

## Publisher’s note

All claims expressed in this article are solely those of the authors and do not necessarily represent those of their affiliated organizations, or those of the publisher, the editors and the reviewers. Any product that may be evaluated in this article, or claim that may be made by its manufacturer, is not guaranteed or endorsed by the publisher.
